# Putative Extracellular Electron Transfer in Methanogenic Archaea

**DOI:** 10.3389/fmicb.2021.611739

**Published:** 2021-03-22

**Authors:** Kailin Gao, Yahai Lu

**Affiliations:** College of Urban and Environmental Sciences, Peking University, Beijing, China

**Keywords:** extracellular electron transfer, methanogenic archaea, c-type cytochrome, archaellum, direct interspecies electron transfer, direct electron transfer

## Abstract

It has been suggested that a few methanogens are capable of extracellular electron transfers. For instance, *Methanosarcina barkeri* can directly capture electrons from the coexisting microbial cells of other species. *Methanothrix harundinacea* and *Methanosarcina horonobensis* retrieve electrons from *Geobacter metallireducens via* direct interspecies electron transfer (DIET). Recently, *Methanobacterium*, designated strain YSL, has been found to grow *via* DIET in the co-culture with *Geobacter metallireducens*. *Methanosarcina acetivorans* can perform anaerobic methane oxidation and respiratory growth relying on Fe(III) reduction through the extracellular electron transfer. *Methanosarcina mazei* is capable of electromethanogenesis under the conditions where electron-transfer mediators like H_2_ or formate are limited. The membrane-bound multiheme c-type cytochromes (MHC) and electrically-conductive cellular appendages have been assumed to mediate the extracellular electron transfer in bacteria like *Geobacter* and *Shewanella* species. These molecules or structures are rare but have been recently identified in a few methanogens. Here, we review the current state of knowledge for the putative extracellular electron transfers in methanogens and highlight the opportunities and challenges for future research.

## Introduction

Methanogens are important to the carbon biogeochemical cycle and global methane emissions. Approximately 1 billion tons of methane is generated annually by methanogens in natural and man-made anoxic environments, as a consequence, about half of that is emitted into the atmosphere (Thauer, 1998; Thauer et al., 2008). Methanogens belong to members of the archaeal domain and occur mostly in the phylum *Euryarchaeota*. The species found to date fall into seven orders that differ both in energy conservation and ecological niches (Liu and Whitman, 2008; Thauer et al., 2008). Methanogenesis can be generally performed through the hydrogenotrophic, aceticlastic, methylotrophic, or methyl-reducing pathways depending on the substrates available in environments. Methanogenesis from syntrophic oxidation of short-chain fatty acids and alcohols is the key process during the anaerobic decomposition of complex organic matter. In this process, H_2_ and formate are usually used as electron transfer mediators. (Stams and Plugge, 2009). An increasing of observations addressing some methanogens perform extracellular electron transfers, however, have questioned this knowledge of methanogenesis.

The extracellular electron transfer (EET) has been well studied in some bacteria regarding *Geobacter* and *Shewanella* species (Shi et al., 2016). EET pathways contain the direct electron transfer (DET) pathway in which solid abiotic materials, such as iron minerals or electrodes, can function as terminal electron acceptors/donors, and the alternative pathway is the direct interspecies electron transfer (DIET) where living microbes can serve as terminal electron acceptors or donors. The strongest evidence for DIET is from a co-culture of *Geobacter metallireducens* and *Geobacter sulfurreducens*, which can oxidize ethanol with the reduction of fumarate to succinate (Summers et al., 2010; Lovley, 2017). DIET or DET cannot be established with the *Geobacter* mutants deprived of the electrically conductive pili (e-pili) or the outer-membrane multi-heme c-type cytochromes (MHC); these components are thus critical to extracellular electron transfer (Summers et al., 2010; Shrestha et al., 2013; Shi et al., 2016; Lovley, 2017).

The DIET pathway has also been proposed in methanogenic aggregates (Morita et al., 2011) and was initially demonstrated in the co-cultures of *G. metallireducens* with *Methanothrix harundinacea* or *Methanosarcina barkeri* (Rotaru et al., 2014a,b). Supplementation of iron oxides (hematite and magnetite) or other conductive materials, such as granular activated carbon, can facilitate the syntrophic growth of co-cultures consuming acetate or ethanol (Kato et al., 2012; Liu et al., 2012). Magnetite-facilitated syntrophic oxidation of propionate and butyrate was detected in methanogenic enrichments (Li et al., 2015; Zhang and Lu, 2016; Xia et al., 2019); consequently, the DIET pathway has been proposed to be an alternate strategy for syntrophic metabolisms in methanogenic environments (Kato et al., 2012; Liu et al., 2012; Li et al., 2015). The discovery of DIET in the co-cultures of *Geobacter* and methanogens (Rotaru et al., 2014a,b; Yee and Rotaru, 2020) supports a long-standing hypothesis that some methanogens can directly obtain electrons from the outside (Dinh et al., 2004). The DET pathways of methanogens have been proposed to occur with electrodes or minerals serving as electron sources (Cheng et al., 2009; Beese-Vasbender et al., 2015; Soo et al., 2016; Yan et al., 2018). Putative DIET and DET in methanogens greatly expand our understanding of methanogen’s roles in biogeochemistry, and future research shall re-assess the contribution of different methanogens to address global methane emission challenges. The mechanisms of extracellular electron transfers from methanogenic archaea, however, have yet to be resolved. The purpose of this review is to summarize the current understanding of putative extracellular electron transfers from diverse methanogens and highlight the challenges of future research.

## Diverse Strategies in *Methanosarcinales*

### Methanosarcina barkeri

*M. barkeri* is metabolically versatile and capable of hydrogenotrophic (H_2_/CO_2_), methylotrophic (methanol, methylamine), methyl-reducing (H_2_ and methanol/methylamine), and aceticlastic (acetate) methanogenesis (Welander and Metcalf, 2005; Thauer et al., 2008; Welte and Deppenmeier, 2014). When performing hydrogenotrophic methanogenesis, *M. barkeri* employs the energy-converting ferredoxin-dependent hydrogenase (Ech), F_420_-reducing hydrogenase (Frh), and membrane-bound methanophenazine-dependent hydrogenase (Vht; Thauer et al., 2008; Kulkarni et al., 2009; Welte and Deppenmeier, 2014). In the pathway of methylotrophic and aceticlastic methanogenesis of *M. barkeri*, Ech, Frh, and Vht also participate (Deppenmeier et al., 1995; Meuer et al., 2002; Welander and Metcalf, 2005; Kulkarni et al., 2009, 2018; Mand et al., 2018). Hydrogenases from *M. barkeri* can catalyze the H_2_ production of electrodes in electrochemical reactors. Cathodes inoculated with *Ms. barkeri*, with the set potential of –0.6 V vs. the standard hydrogen electrode (SHE), could produce H_2_ at a rate of 120 ± 18 nmol d^-1^ ml^-1^, and about half of this rate was detected with the cell extracts of *M. barkeri* (Yates et al., 2014). Recently, it was shown that hydrogenases in combination with ferredoxin (Fd) and F_420_ from *M. barkeri* could attach to the electrode surface and catalyze the formation of H_2_. Then, the produced H_2_ could be consumed rapidly by *M. barkeri*, resulting in a low or undetectable level of H_2_ accumulation (Rowe et al., 2019).

**Figure 1:**
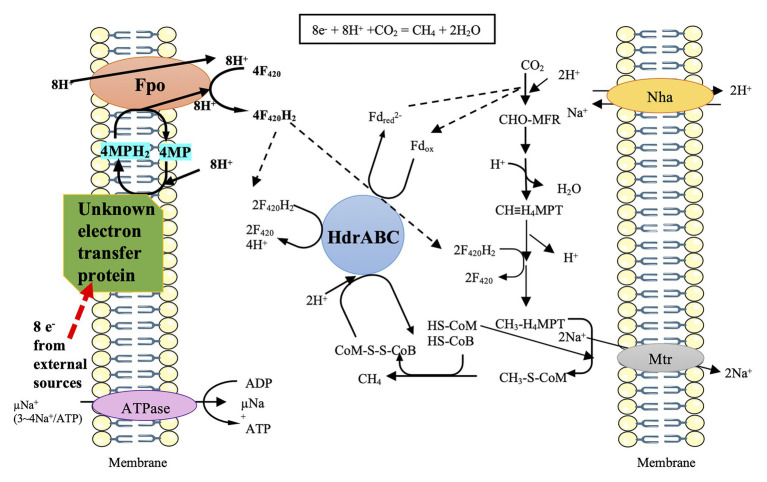
Prediction model of direct electron uptake in *Methanosarcina barkeri*, cited (Holmes et al., 2018). The black arrows represent the possible transfers of electrons *via* F_420_ and Fd. The red arrow represents the possible route for electron uptake from the outside. The unknown electron transfer proteins may gain 8 e^—^ from external sources and then channel these electrons *via* MP/MPH_2_ to Fpo. Fpo can utilize F_420_/F_420_H_2_ to deliver electrons to the process of CO_2_ to CH_4_.

However, a hydrogenase deletion mutant of *M. barkeri* still exhibited the ability of electromethanogenesis with the cathode potential poised at –0.484 V vs. SHE, indicating a hydrogenase-independent mechanism to facilitate the cathodic activity (Rowe et al., 2019). Furthermore, *M. barkeri* is capable of conducting DIET to accept electrons from syntrophic growth with *G. metallireducens* on ethanol (Rotaru et al., 2014a; Holmes et al., 2018). The stoichiometric conversion of ethanol to methane (1.5 CH_4_ per ethanol) in the co-culture of *G. metallireducens* and *M. barkeri* indicated that *M. barkeri* not only metabolized the acetate produced by *G. metallireducens* but also used the electrons released from ethanol oxidation. The transcriptome was compared between the co-culture of *G. metallireducens*/*M. barkeri* and the co-culture of *Pelobacter carbinolicus*/*M. barkeri* (H_2_ was used as the electron transfer mediator) (Holmes et al., 2018). It showed the significant upregulation of gene expression of the most subunits of the membrane-bound F_420_-dehydrogenase (Fpo) in the co-culture of *G. metallireducens*/*M. barkeri*. In addition, the expression of nine genes predicted to be involved in ubiquinone/menaquinone biosynthesis and those genes coding for HdrA_1_B_1_C_1_, HdrA_2_, and HdrB_2_ were also upregulated in the co-culture of *G. metallireducens*/*M. barkeri*. Therefore, a model for the electron and proton flux of the CO_2_ reduction to CH_4_ in *M. barkeri* during DIET-based growth has been postulated based on the above transcriptome comparison (Figure 1; Holmes et al., 2018). *M. barkeri* may obtain electrons from an unknown electron carrier and donate the electrons to methanophenazine (MP), a membrane-bound electron carrier analogous to ubiquinones. Then, the membrane-bound, proton-pumping F_420_-dehydrogenase (Fpo) may transfer electrons from MPH_2_ to F_420_, resulting in the formation of F_420_H_2_. Half of the F_420_H_2_ is proposed to serve as a reductant in the CO_2_ reduction pathway, while the remaining F_420_H_2_ donates electrons to HdrABC. With participation of electron bifurcation, HdrABC may transfer electrons to Fd_ox_ and CoM-S-S-CoB, respectively (Holmes et al., 2018).

*M. barkeri* does not contain MHC, and Fpo has no active sites on the outer surface of the membrane (Welte and Deppenmeier, 2014); how the external electrons are channeled into Fpo thus remains an important question (Figure 1). Future research shall focus on the alternate redox-active proteins in *M. barkeri* that can potentially aid in direct electron uptake. Moreover, a few studies have shown that the membrane-bound methanophenazine greatly contributes to the electrical conductivity of the membrane of *Methanosarcina acetivorans* growing on methanol (Duszenko and Buan, 2017; Yan et al., 2018). Further research is necessary to identify the effect of methanophenazine on the possible augmentation of membrane conductivity in *Ms. barkeri* performing direct electron uptake.

### Methanothrix harundinacea

Both *Methanosarcina* and *Methanothrix* are known as the aceticlastic methanogens, while *Methanothrix* species are the specialists having a much lower threshold concentration for acetate metabolism (Jetten et al., 1992; Welte and Deppenmeier, 2014). *Methanothrix* species have no genes coding for the hydrogenases like Ech, Frh, and Vht (Welte and Deppenmeier, 2011). Although *Methanothrix* species are restricted to acetate degradation, the gene repository for CO_2_ reduction exists in their genome (Rotaru et al., 2014b). Afterwards, *Mt. harundinacea* has been suggested to perform DIET with *G. metallireducens* (Rotaru et al., 2014b; Yee and Rotaru, 2020). The co-culture of *G. metallireducens* and *M. harundinacea* converted ethanol to methane in a stoichiometry of *ca*. 1.5 moles CH_4_ per mole ethanol. The inability of *G. metallireducens* to generate H_2_ or formate and the inability of *M. harundinacea* to metabolize H_2_ or formate ruled out the possibility of electron transfer via mediated electron carrier. So, the finding strongly suggested that DIET occurrence in the co-culture of *G. metallireducens* and *M. harundinacea* (Rotaru et al., 2014b). In addition, the genes coding for CO_2_ reduction were highly expressed in *Mt. harundinacea* from the co-culture (Zhu et al., 2012; Rotaru et al., 2014b). This methanogen, however, cannot utilize the cathode as the sole electron donor (Rotaru et al., 2014b; Yee and Rotaru, 2020).

The specific electron transfer route for DIET remains elusive in *M. harundinacea*. The cell surface of *Methanothrix* genera consists of a protein sheet that is thought to be composed of amyloid proteins. The amyloid proteins can cluster together binding peptides and metal irons, which may facilitate the direct electron uptake from external electron donors (Maji et al., 2009; Viles, 2012; Dueholm et al., 2015; Yee and Rotaru, 2020). However, this is highly speculative and the experimental evidence has yet to be obtained.

### Methanosarcina horonobensis

*M. horonobensis* has a relatively narrow substrate spectrum compared with *M. mazei* and *M. barkeri*, only growing on methanol, dimethylamine, and acetate but not on H_2_/CO_2_ (Shimizu et al., 2011). *M. horonobensis* is able to retrieve electrons from *G. metallireducens via* DIET. Specifically, the co-culture of *G. metallireducens* and *M. horonobensis* converted 8.8 ± 0.4 mM ethanol to 13.1 ± 0.8 mM CH_4_. Therefore, each mole of ethanol yielded *ca*. 1.5 moles of CH_4_, indicating complete conversion of the added ethanol to CH_4_ (Yee et al., 2019). Similar to the co-culture of *M. harundinecea*/*G. metallireducens*, the inability of *G. metallireducens* to generate H_2_ or formate and the inability of *M. horonobensis* to metabolize H_2_ ruled out the possibility of electron transfer via the mediated electron carrier. However, *M. horonobensis* failed to perform electromethanogenesis at the cathode potential poised at –0.4 V vs. SHE (Yee et al., 2019; Yee and Rotaru, 2020). Further research needs to elucidate why *M. horonobensis* can accept electrons from electroactive microbes (typically *G. metallireducens*) rather than from electrodes. *M. horonobensis* has the membrane-bound MHC (Yee and Rotaru, 2020), and future efforts to study DIET of the co-culture of *G. metallireducens* and *M. horonobensis* must be intensified and eventually provide the clarified membrane-bound electron transport chain and MHC expression response to DIET.

### *Methanosarcina mazei* and *Methanothrix soehngenii*

*M. mazei* is closely related to *M. barkeri* in the phylogenetic relationship and can consume a wide range of substrates, including H_2_/CO_2_, methanol, methylamine, and acetate (Welte and Deppenmeier, 2014). It is possible that hydrogenases are coupled with ferredoxin (Fd) and F_420_ from *M. mazei* to capture electrons from cathodes to form H_2_, which is then consumed for the CH_4_ production. However, the experimental evidence for this speculation has yet to be revealed (Welte and Deppenmeier, 2014; Rowe et al., 2019). *M. soehngenii* is a strict non-hydrogenotrophic methanogen. A recent study showed that both *M. mazei* and *M. soehngenii* could pair with *G. metallireducens* (Yee and Rotaru, 2020). However, only 7.7 ± 0.7 mM CH_4_ was produced from 10 mM ethanol in the co-culture of *M. mazei* and *G. metallireducens*, and only 1.8 ± 1.0 mM CH_4_ was produced from 20 mM ethanol in the co-culture of *M. soehngenii* and *G. metallireducens* (Yee and Rotaru, 2020), indicating incomplete conversion of the added ethanol to CH_4_. Therefore, laboratory study should further verify if *M. mazei* and *M. soehngenii* can establish DIET with *G. metallireducens* or other *Geobacter* species.

### Methanosarcina acetivorans

*M. acetivorans* does not possess Ech and Vht hydrogenases and hence is incapable of H_2_-dependent methanogenesis (Ollivier et al., 1984; Sowers et al., 1984; Ferry, 2020). The presence of the membrane-bound Rnf complex (homolog of *rhodobacter* nitrogen fixation complex) can oxidize Fd_red_ or hydroquinone of flavodoxin A (FldA_hq_) to Fd_ox_ or semiquinone of flavodoxin A (FldA_sq_), which distinguishes *M. acetivorans* from all the H_2_-utilizing methanogens among *Methanosarcina* (Li et al., 2006; Wang et al., 2011; Schlegel et al., 2012; Prakash et al., 2019b). It is worth noting that the Rnf genes in *M. acetivorans* cluster with the gene coding for a c-type cytochrome with multiheme-binding motifs (MmcA) (Galagan et al., 2002; Li et al., 2006; Schlegel et al., 2012). The ν *mmcA* mutant strain of *M. acetivorans*, however, still grows on acetate, indicating MmcA is unnecessary for the acetotrophic growth (Holmes et al., 2019).

**Figure 2:**
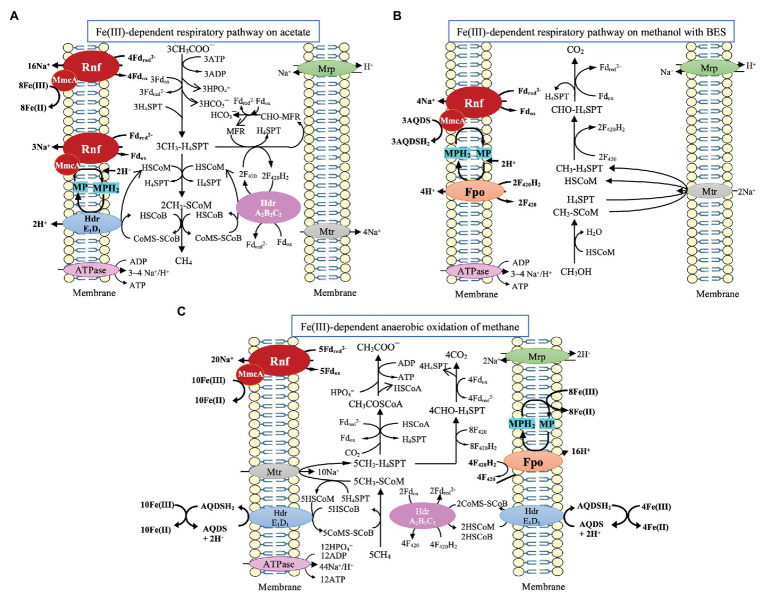
Pathway proposed for Fe(III)-dependent respiratory pathway and CH_4_ oxidation by *Ms. acetivorans*. **(A)** Fe(III)-dependent respiratory pathway on acetate, cited (Prakash et al., 2019a). **(B)** Fe(III)-dependent respiratory pathway on methanol with 2-bromoethanesulfonate (BES), cited (Holmes et al., 2019). **(C)** Fe(III)-dependent CH_4_ oxidation, cited (Yan et al., 2018).

*M. acetivorans* has been shown to perform Fe(III)-dependent respiratory growth on acetate with the simultaneous reduction of Fe(III) to Fe(II) and production of CH_4_. The relevant pathway is illustrated in Figure 2A (Prakash et al., 2019a). One-carbon transformations leading to CH_4_ are the same as its aceticlastic pathway of methanogenesis where Fd_red_ can be generated. By reversal of reactions of the CO_2_ reduction pathway, the respiratory electron transport occurs through oxidation of methyl-tetrahydrosarcinapterin (CH_3_-H_4_SPT) to formyl-methanofuran (CHO-MFR) and then to HCO_3_¯ along with the generation of F_420_H_2_ and Fd_red_ (Lessner et al., 2006; Prakash et al., 2019a; Ferry, 2020). Then, through the combination of Rnf enzyme complex with MmcA, electrons are transferred from Fd_red_ for the reduction of Fe(III) to Fe(II). Two Na^+^ are translocated for each Fe(III) reduced to Fe(II) in this process (Yan et al., 2018; Prakash et al., 2019a). Given that the expression of Fpo is down-regulated, it is postulated that the reoxidation of F_420_H_2_ occurs through the electron bifurcation performing by HdrA_2_B_2_C_2_ (Prakash et al., 2019a; Ferry, 2020). Importantly, the Fe(III)-dependent respiratory growth showed higher acetate consumption, a greater ratio of ATP/ADP, and a higher growth rate, indicating the improved energy conservation (Prakash et al., 2019a; Ferry, 2020). Interestingly, *Ms. acetivorans* can also perform respiratory growth on methanol with AQDS (anthraquinone-2,6-disulfonate, an analog of humic substances in the environment) as the external electron acceptor in the presence of methanogenesis inhibitor 2-biomoethanesulfonate (BES; Figure 2B; Holmes et al., 2019). F_420_H_2_ and Fd_red_ generated from the oxidization of methanol are probably reoxidized by Fpo and Rnf complex, respectively, and electrons are channeled via either MP/MPH_2_ or MmcA for the external reduction of AQDS. Fpo and Rnf can pump H^+^ and Na^+^, respectively. Notably, the ν *mmcA* mutant strain is incapable of methanol-dependent respiratory growth, indicating the importance of MmcA in the external electron transfer (Holmes et al., 2019).

*M. acetivorans* is also capable of Fe(III)-dependent anaerobic oxidation of methane through the reversal of aceticlastic and CO_2_-reducing methanogenesis (Figure 2C; Moran et al., 2005, 2007; Soo et al., 2016; Yan et al., 2018). Methane is assumed to be oxidized by the methyl-coenzyme M methyl reductase (Mcr) to yield methyl-coenzyme M (CH_3_-SCoM), and the methyl group is transferred through Mtr to tetrahydrosarcinapterin (H_4_SPT). During the oxidation of CH_3_-H_4_SPT to CO_2_, Fd_red_ and F_420_H_2_ are formed. Fd_red_ is probably used for the reduction of Fe(III) through the combination of the Rnf complex with MmcA. The Na^+^ gradient generated by the Rnf complex can power the transfer of the methyl group of CH_3_-SCoM to H_4_SPT (Yan et al., 2018). F_420_H_2_ is probably reoxidized by HdrA_2_B_2_C_2_ through electron bifurcation for the coupling reduction of CoM-S-S-CoB and Fd_ox_. The produced HSCoM and HSCoB are postulated to be reoxidized by the membrane-bound HdrE_1_D_1_, driving the AQDS mediated reduction of Fe(III) to Fe(II) (Yan et al., 2018). Additionally, MP is postulated to gain electrons via Fpo from F_420_H_2_, and the produced MPH_2_ is reoxidized for the reduction of Fe(III) (Yan et al., 2018).

Notably, though MHC (also MmcA here) in *Ms. acetivorans* is analogous to MHC in *Shewanella* and *Geobacter* species (Yan et al., 2018; Holmes et al., 2019), the exact route for the MHC-mediated electron transfer has yet to be elucidated. Up to now, there has been no evidence indicating that *Ms. acetivorans* can gain electrons directly from electrodes or materials. It is also unknown whether this methanogen can develop an electrical connection with other microbes like *Geobacter* species. In addition, the deletion of the MHC gene does not obviously impair the growth of *Ms. acetivorans* on acetate and especially does not influence the expression of Rnf genes, indicating MHC gene might be independent of Rnf (Holmes et al., 2019). This independence, however, may promise the flexibility of MHC to interact with other enzymes like Fpo and make *M. acetivorans* adaptable to varying environmental conditions.

## Strategies in Hydrogenotrophic Methanogens

### *Methanospirillum hungatei* With Electrically Conductive Protein Filaments

*Methanospirillum* species belong to the members of *Methanospirillaceae* within the order of *Methanomicrobiales* and represent a group of methanogenic archaea utilizing hydrogen or formate. *Methanospirillum hungatei* JF1 is the first isolated strain of *Methanospirillum* genera (Gunsalus et al., 2016). The potential c-type cytochrome in *M. hungatei* is predicted to be located in the cytoplasm, indicating that it may not take part in the extracellular electron transfer (Yee and Rotaru, 2020). A unique trait of *M. hungatei* is the synthesis of extracellular filaments, called archaella, that can drive cellular motility, promote biofilm formation, and participate in cellular adhesion (Schopf et al., 2008; Jarrell et al., 2011; Jarrell and Albers, 2012; Albers and Jarrell, 2015). The archaella more resemble the bacterial type IV pili in terms of evolution and structure than the bacterial flagella (Faguy et al., 1994; Thomas et al., 2001; Jarrell and Albers, 2012; Albers and Jarrell, 2015, 2018). The type IV pili in the *Geobacter* species have been found to be electrically conductive (hence named e-pili in short), mediating the long-distance extracellular electron transfer (Malvankar et al., 2012; Shi et al., 2016). So far, the e-pili have been explored mainly in bacteria, which raises the question of whether such conductive protein filaments have ever been evolved in archaea.

Initial screening of the relative conductivity of bacterial pili is typically determined with conductive atomic force microscopy (Reguera et al., 2005; Steidl et al., 2016; Sure et al., 2016; Liu et al., 2019), so a similar method was applied to the *M. hungatei* archaella (Walker et al., 2019). To avoid chemical alteration of the archaellum structure and determine the conductivity of hydrated archaella, 100 μl of a culture of *M. hungatei* grown in low-phosphate medium to induce archaellum expression (Faguy et al., 1993) was drop-cast onto highly oriented pyrolytic graphite (HOPG), washed, dried, and equilibrated at 40% relative humidity. Then, a conductive tip serving as a translatable top electrode was used for conductivity measurements. The above process could mimic physiologically relevant conditions (Walker et al., 2019). The local conductive imaging showed that the archaella of *M. hungatei* were electrically conductive. The linear-like current response to applied voltage was revealed in the point-mode current-voltage spectroscopy. The conductance estimated from this response curve was 16.9 ± 3.9 nS for the archaella of *M. hunagtei*, compared with 4.5 ± 0.3 nS for the pili of wild-type *Geobacter sulfurreducens* and 0.004 ± 0.002 nS for the pili of *G. sulfurreducens* strain Aro-5 designed for producing pili with poor conductivity (Reguera et al., 2005; Walker et al., 2019). The atomic model from the cryo-electron microscopy structure of archaella at 3.4 Å resolution revealed that the archaella of *M. hungatei* possessed a core of closely packed phenylalanines (Poweleit et al., 2017). This amino acid arrangement was considered as the key to the electrical conductivity of *M. hungatei* archaella (Walker et al., 2019).

The function of the electrically conductive archaella of *M. hungatei* in nature remains elusive. An earlier study showed that *M. hungatei* could reduce extracellular electron acceptors in which H_2_ was used as the electron-transfer mediator (Cervantes et al., 2002). Electrically conductive archaella of *M. hungatei* may merely facilitate cell attachment by dissipating charge barriers between cells and minerals/electrodes (Walker et al., 2019). Whether the archaella of *M. hungatei* can be used as conduits for the extracellular electron transfer remains an open question. It will be a great challenge to clarify mechanisms of archaellum-mediated electron transport and determine how this electron transport process is coordinated with the catabolic electron flux during growth of *M. hungatei*.

### *Methanococcus maripaludis* Capable of Extracellular Enzyme-Dependent Electron Uptake

*Methanococcus maripaludis*, which belongs to the order *Methanococcales*, is often used as a model for genetic investigation (Leigh et al., 2011). This methanogen utilizes H_2_ and formate as the electron donor to reduce CO_2_ to methane (Brileya et al., 2014). *M. maripaludis* possesses cytoplasmic heterodisulfide reductase (HdrABC) but does not contain membrane-bound HdrDE. The cytoplasmic HdrABC and F_420_-nonreducing hydrogenase (Vhu) form a complex which can perform the flavin-based electron bifurcation, driving the endergonic reduction of oxidized ferredoxin (Fd_ox_) by coupling with the exergonic reduction of heterodisulfide. The reduced ferredoxin (Fd_red_) is used for the first step of CO_2_ reduction by the formyl-methanofuran dehydrogenase (Fwd; Thauer et al., 2008). When formate is supplied, the formate dehydrogenase (Fdh) can be activated and incorporated into the complex of Vhu, Hdr, and Fwd (Thauer et al., 2008; Costa et al., 2010; Kaster et al., 2011). F_420_-reducing hydrogenase (Frh) in the cytoplasm consumes H_2_ to produce F_420_H_2_ feeding electrons into the pathway of methanogenesis (Thauer et al., 2008). Overall, *M. maripaludis* contains six catabolic hydrogenases and an additional energy-converting ferredoxin-dependent hydrogenase (Eha).

**Figure 3:**
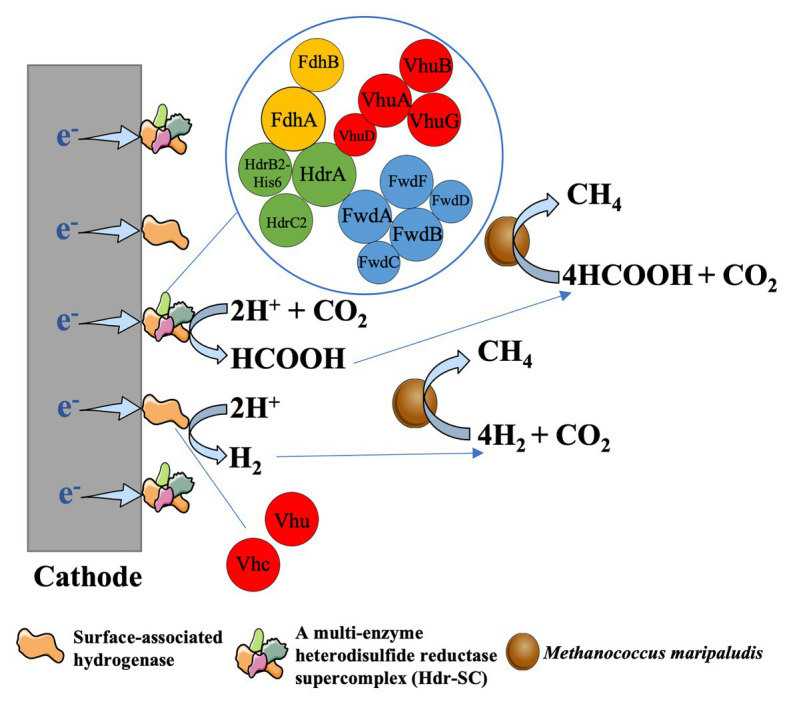
Enzyme-dependent external electron uptake in *Methanococcus maripaludis*, cited (Lienemann et al., 2018). Hydrogenase and formte dehydrogenase can be released from the living or dead cells of methanogens and then are absorbed on the cathode surface. These surface-associated enzymes can catalyze the formation of H_2_ or formate, which was then rapidly consumed by *M. maripaludis* cells to produce CH_4_ (Deutzmann et al., 2015).

Analysis of bioelectrochemical performance revealed that the methane formation rate of the hydrogenase-deprived MM1284 strain of *M. maripaludis* was only about one-tenth of the rate of the wild-type strain (Lohner et al., 2014). The MM1284 mutant carries markerless in-frame deletions of all five catabolic hydrogenase genes except Eha, which is essential to reduce ferredoxin for anabolic reactions (Lie et al., 2012; Costa et al., 2013a; Lohner et al., 2014). The significantly reduced rate of methane production of the MM1284 strain suggested that most of the cathodic electrons used for methane production in the wild type *M. maripaludis* were derived from a hydrogenase-dependent mechanism (Lohner et al., 2014). It was further showed that the hydrogenases and other redox enzymes (like formate dehydrogenases) from cells could precipitate on Fe(0) or the electrode surface. Meanwhile, these enzymes catalyzed the formation of H_2_, or formate, which was then consumed by *M. maripaludis* cells (Deutzmann et al., 2015). In the early view, it was thought that H_2_ or formate was produced from electrodes *via* the abiotic way in the cathode chamber with methanogens. While the above studies showed that H_2_ or formate production was mainly catalyzed by hydrogenase, or formate dehydrogenase from methanogens (biotic way), resulting in the high conversion efficiency of current to methane (Cheng et al., 2009). And this process of electron uptake from electrodes can be defined as surface-associated redox enzyme-dependent electron uptake in methanogens (Figure 3).

The composition of these cell-free and surface-associated redox enzymes however might be complicated. Many studies have shown that hydrogenases and formate dehydrogenases are electroactive and capable of generating hydrogen, or formate by consuming electrons from electrodes (Cracknell et al., 2008; Parkin et al., 2008; Reda et al., 2008; Armstrong et al., 2009; Lojou, 2011; Baffert et al., 2012; Sakai et al., 2016; Chica et al., 2017; Hu et al., 2018; Lienemann et al., 2018; Yuan et al., 2018; Cordas et al., 2019). Recently it has been shown that the *M. maripaludis*-derived NiFeSe hydrogenase and the NiFe hydrogenase, when immobilized at a cathode in a cobaltocene-functionalized polyallylamine (Cc-PAA) redox polymer, could mediate the rapid and efficient hydrogen evolution (Ruth et al., 2020). In addition, a multi-enzyme heterodisulfide reductase supercomplex (Hdr-SC) of *M. maripaludis* was purified. In Fe(0) corrosion experiments, hydrogen formation rates from Fe(0) in the crude lysate amended and purified Hdr-SC vials were 0.14 ± 0.04 μmol d^−1^ μl^−1^ and 0.007 ± 0.03 μmol d^−1^ μl^−1^ lysate equivalent, respectively. The formate formation rate from Fe(0) by cell lysate was 0.62 ± 0.03 μmol d^−1^ μl^−1^, and a quarter of this activity (0.15 ± 0.01 μmol d^−1^ μl^−1^ lysate equivalent) was recovered from purified Hdr-SC (Lienemann et al., 2018). The electrocatalytic activity of purified Hdr-SC was also examined. Upon applying a potential of –0.6 V vs. SHE, the electrochemical reactors with 60 μg of purified Hdr-SC accumulated formate and hydrogen at initial rates of 266 μmol h^−1^ L^−1^ catholyte and 17 μmol h^−1^ L^−1^ catholyte, respectively (Lienemann et al., 2018). Therefore, the hydrogen formation of cell lysate was more likely to be catalyzed by a non-Hdr-SC hydrogenase activity, while Hdr-SC was the main component catalyzing formate production from Fe(0)-derived and cathode-derived electrons. The Hdr-SC in *M. maripaludis* consists of a heterodisulfide reductase (HdrABC), a formate dehydrogenase (FdhAB), and a NiFe-hydrogenase (VhuABDG; Costa et al., 2010, 2013b; Lienemann et al., 2018). In a recent study, homodimeric Hdr complexes containing either (Vhu)_2_ or (Fdh)_2_ have been identified and purified (Milton et al., 2018). Although the structure and function of flavin-based electron bifurcation of Hdr-SC have been documented, it remains unclear why the Hdr-SC deposited on Fe(0) or the cathode surface tends to produce more formate than hydrogen.

It is worth noting that, albeit at a slow rate, the bioelectrochemical methane formation in the hydrogenase-deficient MM1284 strain of *M. maripaludis* was detected (Lohner et al., 2014). Lowering the cathode potential did not increase the rate of methanogenesis, and no formate was detected in the reactors containing MM1284 cells, indicating that a direct electron uptake might occur in the MM1284 strain (Lohner et al., 2014). However, the absence of a detectable level of electron-carrying mediators cannot rule out the possibility of rapid cycling of these redox mediators in the electrochemical reactors. For instance, the cell extracts from MM1284 strain can catalyze formate formation on cathodes (Deutzmann et al., 2015). A recent study designed a combined method using a hydrogen microsensor system and cyclic voltammetry (CV) to determine *in situ* hydrogen concentration within the cathodic biofilm (Cai et al., 2020). A similar method can be explored to detect other *in situ* redox mediators, like formate, within the cathodic biofilm of reactors with MM1284 strain.

## Future Perspectives

*M. barkeri* has been demonstrated to perform DIET and utilize electrons from electrodes. *M. harundinacea* and *M. horonobensis* can retrieve electrons from *G. metallireducens via* DIET but cannot perform electromethanogenesis. *M. acetivorans* can perform Fe(III)-dependent respiratory growth and anaerobic oxidation of methane. *Ms. mazei* is capable of electromethanogenesis under the potential of –0.4 V vs. SHE. A strain of *Methanobacterium*, designated strain YSL, can establish DIET with *G. metallireducens* (Zheng et al., 2020), indicating that the DIET pathway is more broadly distributed among methanogens than previously thought.

However, the external electron-acquisition/donation mechanisms have remained unclear. The external electron-acquisition/donation processes need to coordinate with the internal energy metabolism. Different methanogens perform different energy conservation; as a consequence, the possible external electron-acquisition/donation gadgets may show a high diversity among methanogens. Some methanogens contain MHC, or electrically conductive archaella (*M. hungatei*), while others may have unknown electron-acquisition/donation gadgets.

Many puzzles on the extracellular electron transfer of methanogens remain to be resolved. It warrants further research to figure out why some methanogens can accept electrons from other microbes but cannot utilize electrons from electrodes. Additionally, it remains unclear whether the electrically conductive archaella of *M. hungatei* can help *M. hungatei* electrically interact with other microbes, minerals, or electrodes. It is also unknown whether *M. acetivorans* can gain electrons directly from the outside. Moreover, given the diversity of *Methanosarcina* species, different kinds of external electron-acquisition/donation gadgets may exist and are worthy to be further explored. The novel technologies, such as metagenomics, metatranscriptomics, and high-resolution cryo-electron microscopy, may help us identify more methanogens that may perform DIET or DET in nature methanogenic communities. The biochemical approaches, for example, using the washed everted membrane vesicles to study Fe(III)-dependent anaerobic oxidation of methane in *M. acetivorans* (Yan et al., 2018), can also be employed to explore the mechanisms of DIET/DET in other methanogens.

## Author Contributions

KG and YL conceived the study and wrote the manuscript. All authors contributed to the article and approved the submitted version.

### Conflict of Interest

The authors declare that the research was conducted in the absence of any commercial or financial relationships that could be construed as a potential conflict of interest.
